# Glia maturation factor beta deficiency protects against diabetic osteoporosis by suppressing osteoclast hyperactivity

**DOI:** 10.1038/s12276-023-00980-8

**Published:** 2023-05-01

**Authors:** Si Shi, Huijie Gu, Jinyuan Xu, Wan Sun, Caiyin Liu, Tong Zhu, Juan Wang, Furong Gao, Jieping Zhang, Qingjian Ou, Caixia Jin, Jingying Xu, Hao Chen, Jiao Li, Guotong Xu, Haibin Tian, Lixia Lu

**Affiliations:** 1 Department of Ophthalmology of the Shanghai Tongji Hospital Affiliated with Tongji University, School of Medicine, and Tongji Eye Institute, 389 Xinchun Road, Shanghai, 200065 PR China; 2 Department of Orthopedics, Minhang Hospital, Fudan University, 170 Xinsong Road, Shanghai, 201199 PR China; 3 Department of Ophthalmology of Ten People Hospital Affiliated with Tongji University, School of Medicine, Shanghai, 200072 PR China; 4 Department of Pharmacology, Tongji University School of Medicine, Shanghai, PR China; 5 Department of Biochemistry and Molecular Biology, School of Medicine, Tongji University, 1239 Siping Road, Shanghai, 200092 PR China

**Keywords:** Type 1 diabetes, Diabetes complications

## Abstract

Excessive osteoclast activation, which depends on dramatic changes in actin dynamics, causes osteoporosis (OP). The molecular mechanism of osteoclast activation in OP related to type 1 diabetes (T1D) remains unclear. Glia maturation factor beta (GMFB) is considered a growth and differentiation factor for both glia and neurons. Here, we demonstrated that Gmfb deficiency effectively ameliorated the phenotype of T1D-OP in rats by inhibiting osteoclast hyperactivity. In vitro assays showed that GMFB participated in osteoclast activation rather than proliferation. Gmfb deficiency did not affect osteoclast sealing zone (SZ) formation but effectively decreased the SZ area by decreasing actin depolymerization. When GMFB was overexpressed in Gmfb-deficient osteoclasts, the size of the SZ area was enlarged in a dose-dependent manner. Moreover, decreased actin depolymerization led to a decrease in nuclear G-actin, which activated MKL1/SRF-dependent gene transcription. We found that pro-osteoclastogenic factors (Mmp9 and Mmp14) were downregulated, while anti-osteoclastogenic factors (Cftr and Fhl2) were upregulated in Gmfb KO osteoclasts. A GMFB inhibitor, DS-30, targeting the binding site of GMFB and Arp2/3, was obtained. Biocore analysis revealed a high affinity between DS-30 and GMFB in a dose-dependent manner. As expected, DS-30 strongly suppressed osteoclast hyperactivity in vivo and in vitro. In conclusion, our work identified a new therapeutic strategy for T1D-OP treatment. The discovery of GMFB inhibitors will contribute to translational research on T1D-OP.

## Molecular genetics: RNA splicing in health and disease

Cell signaling processes are affected by the varying ways that sections of messenger RNA (mRNA), the molecule that carries genetic instructions copied from a gene, are spliced together to generate several different proteins from a single gene. Kee K. Kim et al. at Chungnam National University in Daejeon, South Korea, review the significance for cell signaling of the alternative splicing of mRNAs. Mutations or abnormal expression of splicing factors that lead to the splicing processes going awry are implicated in human diseases, including cancer. The authors examine the most recent research insights gained by applying emerging methods of genetic analysis to the role of mRNA splicing in several specific signaling pathways vital for normal development and health. They suggest that increasing understanding of faulty splicing in disease could open avenues toward new forms of treatment.

## Introduction

Osteoporosis (OP) secondary to type 1 diabetes (T1D) is a lesser-known complication but is receiving increasing attention due to the better medication for and longer lifespan of patients with T1D^1^. Patients with T1D have a 2–7-fold higher risk of osteoporotic fracture^2^. An imbalance between osteoblasts and osteoclasts is involved in the pathogenesis of T1D-OP^3^.

However, the role of osteoclasts in T1D-OP is controversial, with reports showing no change^4^, a decrease^5^, or an increase in the number and activity of osteoclasts^6–10^, partly due to the different OP models used, time points, and severity of T1D-OP. Some studies revealed an elevated level of bone-resorbing markers (such as Nfatc1, Ctsk, and TRACP-5b)^7–9^ and pro-osteoclastogenic cytokines (IL-1, IL-6, IL-7, and IL-17a)^10^. In addition, clinically, antiresorptives, such as bisphosphonates, can prevent bone loss in T1D-OP patients^11^. This evidence suggests hyperactivity of osteoclasts in T1D-OP.

A previous report showed that the formation of unique cell adhesion structures, called the sealing zone (SZ) or actin ring, is essential to the activation of bone resorption for mature osteoclasts^12^. These SZs are required for the dynamics of actin polymerization and depolymerization, which are controlled by several actin-binding proteins (such as gelsolin^13^) and the Arp2/Arp3 complex^14^ and Arp2/Arp3 complex-related proteins (Cdc42^15^, Cortactin^16^, and WASP^17^). Thus, we proposed that the dynamics of actin in osteoclasts may represent a promising therapeutic target for T1D-OP.

GMFB has recently been identified as an actin cytoskeletal regulator, particularly in remodeling actin network architecture. GMFB is one of the five actin-regulating proteins belonging to the ADF-H family that is conserved from yeast to mammals. GMFB has a high affinity for the Arp2/3 complex but does not bind actin. By interacting with the Arp2/3 complex, GMF facilitates the debranching of actin filament networks and prevents actin nucleation^18^. However, the function of GMFB in T1D-OP is unclear.

Numerous studies have focused on the role of GMFB in neuroinflammation and neurodegeneration since it was primarily isolated from astrocytes^19^. GMFB induces interleukin (IL)-33 release from mouse astrocytes, which in turn augments the release of tumor necrosis factor (TNF)-α and other proinflammatory cytokines/chemokines from these astrocytes^20^. GMFB expression is also upregulated in Alzheimer’s disease^21^ and Parkinson’s disease^22^. Our previous work showed that GMFB, as an early mediator in diabetic retinopathy (DR), promoted ferroptosis in retinal pigment epithelial cells^23^. Based on the roles of GMFB in actin dynamics and DR, we hypothesized that GMFB participates in T1D-OP and that targeting GMFB might be a novel strategy for the treatment of T1D-OP.

In the present study, taking a hypothesis-driven approach, for the first time, we explored the role of GMFB in diabetic osteoclasts in vivo and in vitro. We found that GMFB was upregulated during osteoclast maturation. Gmfb knockout (KO) alleviated OP in the T1D rat model and inhibited SZ formation by regulating actin dynamics. Rescue experiments indicated that GMFB was essential for SZ enlargement under hyperglycemic conditions. We further showed that the GMFB/G-F actin/MKL1 axis regulated the hyperactivity of osteoclasts. Finally, a small-molecule inhibitor for GMFB, DS-30, was screened and validated for its antiosteoporotic activity in rats with T1D-OP. Targeting GMFB in T1D-OP patients merits clinical investigation.

## Materials and methods

### Animal models

All animal experiments were approved by the Tongji University Committee of Experimental Animal Ethics (Permit Number: TJAA09620207), Shanghai, China. All procedures involving animals were carried out following the National Institutes of Health Guide for the Care and Use of Laboratory Animals (NIH Publication No. 8023, revised 1978). Gmfb knockout Sprague‒Dawley (SD) rats were generated by the CRISPR‒Cas9 technique. In detail, a single base A insertion at the 14th aa in exon 2 caused a frame shift, leading to premature termination at 37 aa in exon 2. Finally, nonfunctional truncated GMFB was produced, herein referred to as GMFB−/−. The rats were housed and maintained in the specific pathogen-free (SPF) facility of Tongji University, Shanghai, on a 12–12 h day-night cycle with ad libitum access to water and food. Streptozocin (STZ)-induced diabetic SD rats were prepared as previously described^23^. In brief, all male SD rats weighed ~180 g at the time of intraperitoneal (i.p.) injections of STZ (60 mg/kg, HY-13753, MedChemExpress, Monmouth, NJ, USA) after fasting for 12–24 h. Diabetes was determined using a drop of blood from the tail vein when blood glucose exceeded 250 mg/dl for 3 consecutive days. The candidate GMFB inhibitor DS-30 was administered daily at 50 μM i.p. on the first day of modeling for 4 weeks.

### Microcomputed tomography analysis

The proximal tibia was isolated and fixed with 4% paraformaldehyde (PFA) overnight. Then, proximal tibias were scanned using the Skyscan-176 micro-CT system (Bruker micro-CT, Belgium) at a voxel resolution of 8.96 μm. Bone parameters were measured according to our previous report^24^. Two-dimensional (2D) images and three-dimensional (3D) images were obtained by MIMICS software. The analysis of BMD, BV/TV, Tb. N, and Tb. Sp was performed by CTan (Bruker) for visualization and analysis of volumetric image data.

### Bone histomorphometric analysis

Histomorphometric analysis was performed using our previously established protocol^24^. For dynamic histomorphometry, all the rats were given two i.p. injections of calcein (15 mg/kg, C0875, Sigma-Aldrich, St. Louis, MO, USA) on the 10th day and 3rd day before being euthanized. The proximal tibiae were fixed in 70% ethanol and embedded without decalcification in polymethyl methacrylate (MMA) resin for histological sectioning. Sections were obtained from the tibiae at a thickness of 5 μm with a Leica SM2500E microtome and photographed at a 1 mm^2^ area within the metaphyseal secondary spongiosa, originating 1 mm below the growth plate using Nikon ECLIPSE Ti (Shinagawa, Japan). Bone dynamic histomorphometric analyses were performed using ImageJ (version 1.53t; Bethesda, MD).

### Histology and immunohistochemistry

Tibias were fixed in 4% PFA at 4 °C for 24 h and then decalcified in 10% EDTA. Fixed samples were processed for dehydration, clearing, infiltration, and embedded in paraffin. Proximal tibial Section (5 μm) (RM2235, Leica, Solms, Germany) were stained with hematoxylin and eosin (HE) (H9627, Sigma-Aldrich) to visualize adipocytes (diameter greater than 30 μm) and stained with osteocalcin (OCN) (23418-1-AP, Proteintech, Chicago, USA) to display the osteoblasts per field. All histomorphometric analyses of each group were performed using ImageJ software.

### In vivo tartrate-resistant acid phosphatase (TRAP) staining

Decalcified proximal tibial Section (5 μm) (RM2235, Leica) were stained with TRAP staining buffer (387 A, Sigma) to visualize the osteoclasts (measured ranging from the proximal growth plate to 2 mm distal) per field. All histomorphometric analyses of each group were performed using ImageJ software.

### Osteoclast differentiation and TRAP staining

Osteoclasts were generated as previously described^25^. Briefly, adherent osteoclast precursors were incubated in α-MEM complete medium with 25 ng/ml recombinant rat M-CSF (25 ng ml^−1^) (AF-400-28-10, Peprotech, NJ, USA) at 37 °C with 5% CO_2_ for 3 days followed by 4 days of treatment with M-CSF and RANKL (50 ng ml^−1^) (9366-TN, R&D, Minneapolis, MN, USA) for the induction of osteoclast maturation. Additional glucose (25 mM) was added on day 5 of differentiation. A GMFB candidate inhibitor was added on day 3 of differentiation. Cytochalasin B was added to osteoclasts at a final concentration of 1 μM for 24 h or 10 μM for 10 min before harvesting. The MKL1/SRF inhibitor CCG-1423 (285986-88-1, MedChemExpress) was added to osteoclasts at a final concentration of 1 μM for 24 h before harvesting. TRAP staining was carried out, and the number of TRAP-positive cells (multinucleated, large, spread) containing more than 3 nuclei/cell was counted.

### Bone resorption assay

Resorption assays were performed using Osteo Assay Surface plates (3989, Corning, NY, USA), which mimic in vivo bone. Cells were seeded at a density of 1 × 10^4^ cells/well and cultured in osteoclastogenic differentiation medium as previously described. Then, the mature OCs were washed away by incubation with 5% sodium hypochlorite solution for 30 min, washed with ddH_2_O three times and air dried for 2 h. Resorption pits were observed under an optical microscope (Nikon ECLIPSE Ti, Japan), and the pit area was analyzed by ImageJ software.

### Cell proliferation assay

Osteoclast cell proliferation was determined by the BeyoClick™ 5-ethynyl-2’-deoxyuridine (EdU) Cell Proliferation Kit with TMB (C0088, Beyotime, Shanghai, China) according to the manufacturers’ protocol. Each well was then developed with TMB substrate and read at 370 nm. This experiment was repeated three times.

### Transient transfection

Human Gmfb was cloned into the pcDNA3.1 vector, and 0.5 μg of pcDNA3.1-hGmfb was transfected into osteoclasts on day 4 of differentiation using Lipofectamine^TM^ 3000 Reagent (L3000150, Thermo Fisher Scientific, Waltham, MA, USA) according to the manufacturer’s instructions. Cells were incubated for 24 h before further analyses.

### Immunocytochemical staining

For immunocytochemical staining, the fixed mature osteoclasts were incubated with primary antibodies against Arp2 (1:50, D221703, BBI, Sangon Biotech, Shanghai, China), GMFB (1:50, SP-61, Santa Cruz, CA, USA), DBP (1:150, D163666, BBI), DNase I (1:100, D222246, BBI), or MKL1 (1:50, sc-398675, Santa Cruz) at 4 °C overnight, followed by Alexa 647- and Alexa 488-conjugated secondary Abs (1:1000, abcam, Cambridge, UK) for 1 h. Images were taken on a Leica TCS SP5 confocal scanning laser microscope (Leica Laser Technik, Solms, Germany).

### SZ formation assay

Primary osteoclasts were cultured on glass coverslips. Fluorescent labeling was performed with TRITC phalloidin (200 nM, 40734ES80, Yeasen, Shanghai, China) to determine actin organization. Images were captured using a Leica TCS SP5 confocal scanning laser microscope. The relative size of the SZ in each group was measured using ImageJ software.

### Enzyme-linked immunosorbent assay (ELISA)

Serum was collected by centrifugation in 2 ml Eppendorf tubes at 2000 × *g* for 10 min at 4 °C. Serum levels of indices of bone resorption were determined by rat ELISA kits for CTX-1 and TRACP-5b according to the manufacturer’s instructions (XunQing Biotech Company, Shanghai, China).

### Quantitative reverse transcription-polymerase chain reaction

Total RNA was isolated using TRIzol reagent (10296010, Invitrogen, Carlsbad, CA, USA). cDNA was synthesized using PrimeScript RT polymerase (RR036A, TaKaRa, Kusatsu, Shiga Japan) according to the manufacturer’s instructions. Amplification reactions were set up in duplicate using SYBR Green Premix (FP205, Tiangen, Beijing, China) with the following cycling parameters: denaturation at 95 °C for 15 min, followed by 40 cycles of 95 °C for 30 s and 60 °C for 30 s. Primer sequences are listed in Supplementary Table 1.

### Western blot analysis

Cells were lysed with radioimmunoprecipitation assay (RIPA) buffer (P0013B, Beyotime, Shanghai, China) containing protease inhibitors and phosphatase inhibitors (C0001 and C0004, TargetMol, Massachusetts, USA). Total lysates were separated by sodium dodecyl sulfate‒polyacrylamide gel electrophoresis (SDS‒PAGE, C671102, Sangon Biotech) and transferred to polyvinylidene fluoride (PVDF) membranes (IPVH00005, Millipore, Burlington, MA, USA). The membranes were incubated with GMFB-specific antibodies (1:200, SP-61, Santa Cruz) and visualized with appropriate horseradish peroxidase (HRP)-conjugated secondary antibodies (1:10,000). The corresponding bands were detected using an enhanced chemiluminescent (ECL) detection kit (36208ES60, Yeasen).

### Measurement of the G/F actin ratio by Triton X-100 fractionation and amido black staining

G/F actin fractionation was performed according to the protocol Zeng reported previously^26^. The osteoclasts were grown in 24-well plates for subsequent fractionation. Western blotting was carried out with fractionation using a monoclonal rabbit anti-pan-actin-HRP conjugated antibody (1:1000, #12748, CST, Cambridge, MA, USA). The PVDF membrane was stained with amido black for 5 min to visualize the total protein band, which was used as a loading control as previously described^27^. Grayscale scanning and quantification were performed using ImageJ.

### Screening and identification of small-molecule inhibitors for GMFB

Based on the binding site between GMFB and Arp2/3^28^, computerized virtual screening for potential GMFB inhibitors was conducted by the TargetMol Chemicals Inc with the ChemDiv compound library. Biocore analysis was performed to determine the binding affinity between GMFB and candidate inhibitors by Chen Hongzhuan’s laboratory (Shanghai Jiaotong University).

### Cell viability assay

A CCK-8 assay was used to evaluate the cytotoxicity of candidate inhibitors. In brief, cells (including primary osteoclasts, HEK293T cells, and 3T3-L1 cells) were seeded in 96-well plates for 24 h and treated using candidate inhibitors at different concentrations (osteoclasts were treated with DS-30 on day 3 of differentiation for 3 days). After a 24 h treatment, the cells were incubated with 10% CCK-8 solution(C0005, TargetMol) for 1 h at 37 °C, and then, the absorbance at 450 nm was measured. Data from at least three sets of samples were used for statistical analysis.

### Statistical analysis

All experiments were performed in triplicate, and all data are expressed as the mean ± SEM, except where noted. Comparisons between two groups were performed using an unpaired Student’s *t* test, while comparisons of multiple groups were performed using one-way ANOVA with Prism 8 software. A *p* value less than 0.05 was considered significant.

## Results

### GMFB KO protected against OP in rats with T1D

**Fig. 1:**
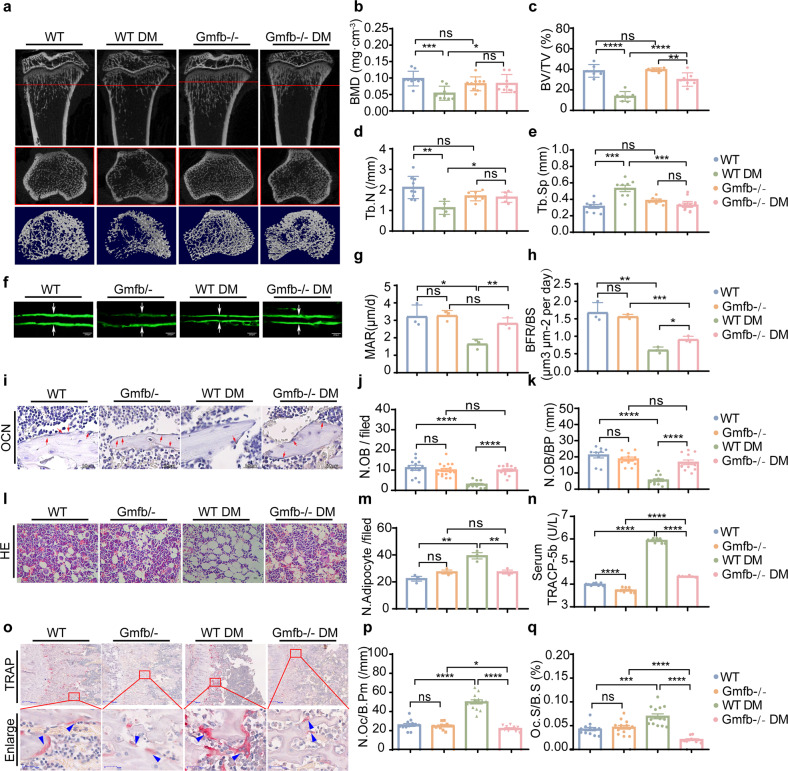
Gmfb KO attenuated the osteoporotic phenotype and decreased excess bone resorption in T1D-OP in vivo. **a** Representative micro-CT 3D reconstruction images of proximal tibias. Quantitative analysis of bone morphology, including bone mineral density (BMD) (**b**), bone volume per total volume (BV/TV) (**c**), mean trabecular number (Tb. N) (**d**), and mean trabecular separation (Tb. Sp) (**e**) in tibia from the different groups. Each point represents one rat. *n* = 7–10. Data are expressed as the mean ± SD. **f**–**h** High-magnification calcein double labeling of sections of the tibia of the WT control and GMFB−/− rats 4 weeks after STZ treatment (or no treatment). The distance between two arrows represents a width of 7 days of new bone formation. Dynamic histomorphometric analysis of MAR (**g**) and BFR/BS (**h**) analyzed by double calcein labeling. Scale bars, 50 μm. *n* = 3. **i**–**k** The changes in osteoblasts were observed by immunohistochemical staining against OCN. Bone histomorphometric analysis of trabecular bone among each group, including osteoblast number per field (N.Ob/per field; **j**) and number of osteoblasts per bone surface (N.Ob/B. Pm; **k**). Scale bars, 50 μm. Red arrows indicate osteoblasts. *n* = 3. **l**, **m** Tibial section in (**l**) stained by hematoxylin and eosin (representative images), scale bars, 10 μm. The number of adipocytes was measured using ImageJ (**m**). *n* = 3. **n** Serum concentrations of TRAP5b detected in the WT and Gmfb KO rats with or without STZ by ELISAs. Data are presented as the mean ± SD of three independent experiments. **o**–**q** TRAP staining of the proximal tibial section was performed to visualize the activated osteoclasts (**o**). Red arrowheads indicate osteoclasts. Bone histomorphometric analysis for the number of osteoclasts per bone perimeter (N.Oc/B. Pm, **p**) and osteoclast surface per bone surface (OC.S/BS, **q**) in the proximal tibia. Scale bars, 100 μm (upper) and 20 μm (below). Each point represents one cell. *p < 0.05, **p < 0.01, ***p < 0.0001 and ****p < 0.00001 (Student’s *t* test).

First, GMFB KO rats were generated using CRISPR/Cas9 technology by targeting exon 2 of Gmfb to obtain a +1 mutation in exon 2 (Supplementary Fig. 1a, b). The Gmfb KO rats were born normally and had little difference in size, weight, growth, or fecundity compared to the control littermates (Supplementary Fig. 1c, d). We first measured the bone mass to confirm the onset of T1D-OP by Micro-CT (Supplementary Fig. 1e–g and Fig. 1a–e). Micro-CT of the proximal tibiae showed that there was no significant difference between the wild-type (WT) group and the KO group in terms of bone mineral density (BMD), trabecular number (Tb. N), trabecular separation (Tb. Sp), and bone volume/tissue volume ratio (BV/TV) (Fig. 1a–e). At 4 weeks after T1D onset, the diabetic rats exhibited an osteoporotic bone phenotype. However, Gmfb KO significantly increased BMD, Tb. N, and BV/TV while reducing Tb. Sp under diabetic conditions (Fig. 1a–e), indicating that Gmfb KO protected against T1D-OP.

The calcein double-labeling experiment confirmed that the impaired bone formation and mineral apposition rate caused by T1D was restored in the Gmfb−/− rats (Fig. 1g, h). The higher OCN^+^ cell numbers in the Gmfb−/− tibias than in the wild-type tibias under diabetic conditions indicated more osteoblast bone formation (Fig. 1i–k). Furthermore, HE staining showed that deletion of Gmfb led to relatively lower marrow adiposity, which is a hallmark of patients with T1D^29^ (Fig. 1l, m).

Since the role of osteoclasts in T1D-OP is controversial, we investigated whether bone resorption was involved in the changes in bone phenotype in Gmfb KO rats in a diabetic context. Serum TRACP-5b and CTX-1 were measured to assess systematic osteoclast activity. A significantly elevated serum level of TRACP-5b in the diabetic WT group was suppressed by Gmfb deficiency (Fig. 1n), whereas the CTX-1 level remained unchanged (Supplementary Fig. 1h). In addition, TRAP+ osteoclasts in the WT rats exhibited a flattened shape, while those of the KO rats exhibited a smaller, rounded shape and occasionally a flattened shape under diabetic conditions (Fig. 1o). The numbers of TRAP+ osteoclasts (N.Oc/B. Pm) and osteoclast surface per bone surface (Oc. S/BS) in histological sections of Gmfb KO tibial bone were substantially decreased (Fig. 1p, q). These data indicated that the diabetic-induced osteoporotic bone phenotype was partially recovered in the Gmfb KO rats by inhibition of excessive bone resorption.

### GMFB mediated osteoclastogenesis

**Fig. 2:**
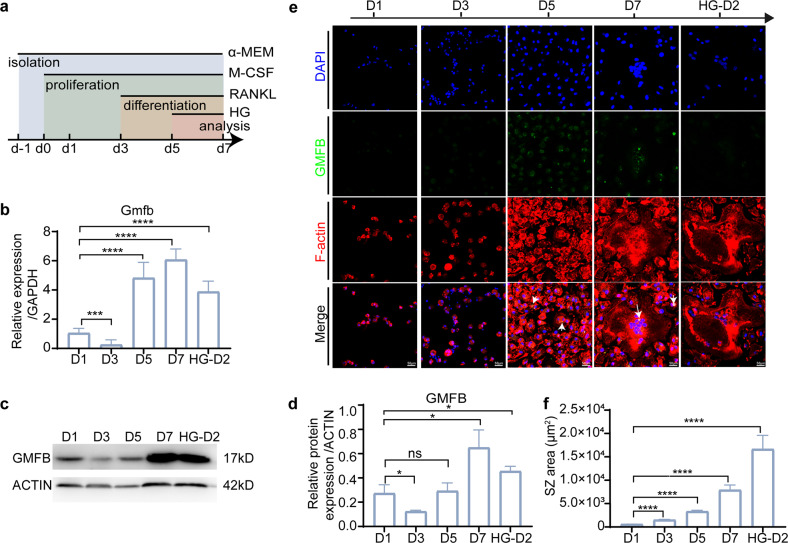
GMFB participated in the differentiation and maturation of osteoclasts. **a** A schematic illustration of the schedule for primary osteoclast culture. **b** Gmfb mRNA concentrations were determined by RT‒qPCR on d1, d3, d5, and d7 of differentiation and normalized to GAPDH mRNA. Data are presented as the mean ± SD of three independent experiments. **c** WB analysis of GMFB in osteoclasts on d1, d3, d5, and d7 of differentiation. **d** Quantitative analyses of (**c**). Data are presented as the mean ± SD of three independent experiments. **e** Osteoclasts from the same experiment in (**a**–**d**) cultured on round cover glasses were immunostained for F-actin (red, phalloidin), GMFB (green), and nuclei (blue, DAPI). Scale bar: 100 μm. **f** Quantitative analysis of SZ area during osteoclastogenesis in (**e**). *p < 0.05, **p < 0.01, ***p < 0.0001 and ****p < 0.00001 (Student’s *t* test).

Given that Gmfb KO suppressed the hyperactivity of osteoclasts under diabetes, we investigated whether GMFB participates in osteoclastogenesis. To this end, we first examined the GMFB expression pattern during osteoclastogenesis. A classic protocol with M-CSF and RANKL treatment for osteoclast induction was used in this study (Fig. 2a). On day 3 with single M-CSF treatment, significant downregulation of GMFB in primary osteoclasts was observed, while notable upregulation of GMFB was found in response to RANKL treatment at day 5 and day 7 (Fig. 2b–d). In parallel, we observed that increased F-actin stress fibers gradually transformed into a complete actin-rich ring-like SZ (Fig. 2e, f). During osteoclastogenesis, the SZ area increased over time. GMFB was found to partially colocalize with F-actin (Fig. 2e). After high glucose (HG) treatment for 2 days (HG-D2), a high level of GMFB was maintained, and a clearly larger SZ formed in osteoclasts. Thus, the differential expression of GMFB in response to M-CSF and RANKL treatment suggested an important role of GMFB in osteoclastogenesis.

### Gmfb KO promoted the production of osteomorphs and partially inhibited osteoclast activity in vitro

**Fig. 3:**
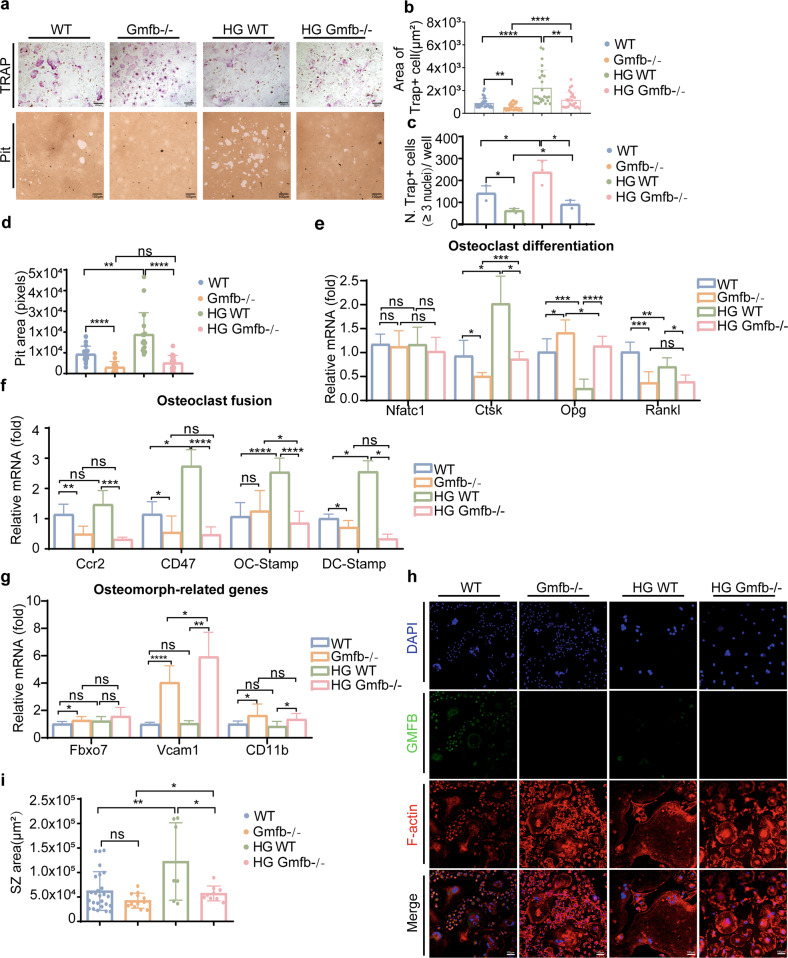
Gmfb KO osteoclasts are defective in SZ formation. **a** Osteoclasts were seeded on osteologic substrate to show TRAP activity (upper lane) and osteoclast resorption activity (below, quantified by assay of pit area) in cultures with M-CSF and RANKL for 7 days. Quantitative analysis of the area (**b**) and number (**c**) of TRAP+ cells on day 7 of osteoclastogenesis in (**a**). **d** The pit resorption area was quantified to assess osteoclast resorption activity. Each point represents one cell. *n* = 10–15. **e** Detection of the osteoclast differentiation-related genes Nfatc1, Ctsk, Opg, and Rankl by RT‒qPCR. mRNA levels were normalized to Gadph mRNA levels. Data are presented as the mean ± SD of three independent experiments. **f** Detection of the osteoclast fusion regulatory genes Ccr2, CD47, OC-Stamp, and DC-Stamp by RT‒qPCR. mRNA levels were normalized to Gadph mRNA levels. **g** Detection of the osteomorph-related genes Fbxo7, Vcam1, and CD11b by RT‒qPCR. mRNA levels were normalized to Gadph mRNA levels. Data are presented as the mean ± SD of three independent experiments. **h** Osteoclasts from the same experiment in (**a**) cultured on round cover glasses were immunostained for F-actin (red, phalloidin), GMFB (green), and nuclei (blue, DAPI). Scale bar: 100 μm. **i** Quantitative analysis of the SZ area on day 7 of osteoclastogenesis in (**f**). Each point represents one cell. *n* = 7–26. Data are expressed as the mean ± SD. *p < 0.05, **p < 0.01, ***p < 0.0001 and ****p < 0.00001 (Student’s *t* test).

Next, we examined the effect of Gmfb KO on osteoclasts. A significant decrease in the TRAP+ area of Gmfb KO osteoclasts was observed. Consistent with our findings in vivo (Fig. 1o), the HG-induced increase in the area and number of multinucleated TRAP+ cells (≥3 nuclei) from the WT rats was significantly suppressed in the Gmfb KO osteoclasts (Fig. 3a–c). Resorption pits resorbed by the Gmfb KO osteoclasts were smaller and shallower than those resorbed by the WT cells, indicating a negative regulation of osteoclastic bone resorption (Fig. 3a, d). These results confirmed that GMFB deficiency inhibited osteoclast activation.

NFATc1, which drives osteoclast proliferation^30^, remained unchanged among the groups, while mature osteoclast markers (including Ctsk and Rankl) were downregulated when Gmfb was deficient, indicating that GMFB did not affect early osteoclast commitment but functioned in late differentiation (Fig. 3e). However, we did not find a significant difference in the proliferation rate between the WT and Gmfb KO osteoclasts, which was consistent with the qPCR results (Supplementary Fig. 2a). Moreover, Gmfb deficiency inhibited the generation of large polykaryons, which developed through osteoclast fusion. The downregulation of osteoclast fusion-related markers (CCR2, CD47, OC-Stamp, and DC-Stamp) further suggested that Gmfb KO inhibited cell fusion (Fig. 3e). After complete induction and differentiation of osteoclasts, more osteomorphs, a new cell type of small osteoclasts, were observed in the Gmfb KO osteoclasts than in the WT osteoclasts (Fig. 3a). Vcam1, Fbxo7, and CD11b have been identified as molecular signatures for osteomorphs^31^. qRT‒PCR was performed to measure the expression of these genes. We found that compared to those in the WT osteoclasts, the osteomorph-associated marker genes CD11b and Vcam1 were upregulated in the Gmfb KO osteoclasts, except for Fbxo7, which indicated that Gmfb KO inhibited RANKL-dependent fusion, resulting in the accumulation of osteomorphs (Fig. 3g). Downregulation of osteoclast fusion- and maturation-related genes and upregulation of osteomorph genes in the Gmfb KO osteoclasts may contribute to the limited activity of osteoclasts. Morphologically, we observed that SZ formation with a smaller size occurred in the Gmfb KO osteoclasts, but the larger SZ induced by HG treatment in the WT osteoclasts did not appear in the Gmfb KO osteoclasts (Fig. 3h, i). Conversely, overexpression of GMFB in the Gmfb KO osteoclasts resulted in significant enlargement of SZ formation (Supplementary Fig. 2b, c). In summary, we show that Gmfb KO produced more osteomorphs and suppressed osteoclast hyperactivation by inhibiting osteoclast fusion and limiting SZ formation under HG treatment.

### Gmfb deficiency negatively regulated SZ formation by decreasing the G/F-actin ratio

**Fig. 4:**
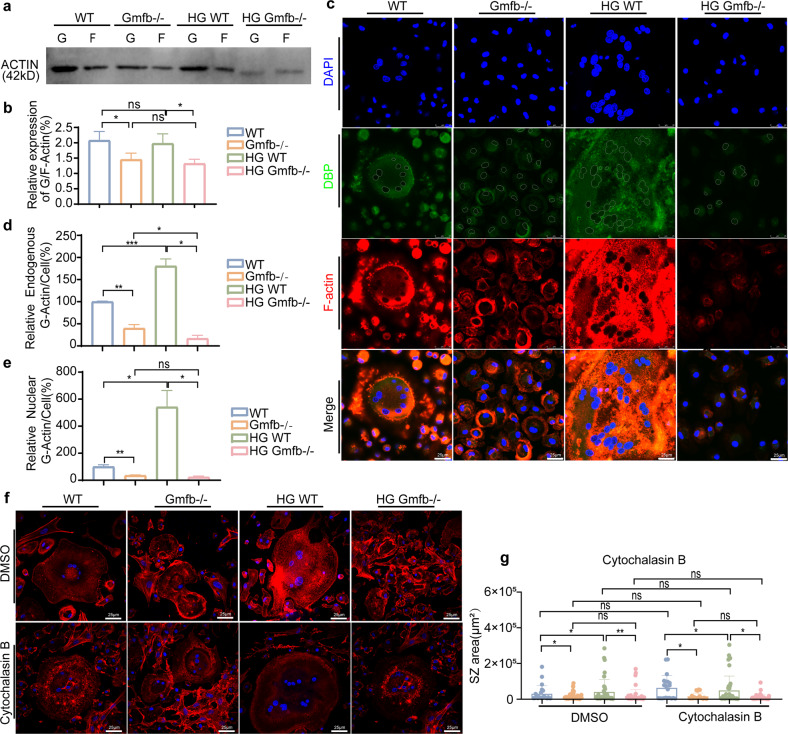
Gmfb deficiency negatively regulated SZ formation by decreasing the G/F-actin ratio. **a**, **b** Western blot quantification of the G/F actin ratio within cells with or without HG. Data are presented as the mean ± SD of three independent experiments. **c** Osteoclasts treated with differentiation medium for 7 days were immunostained for F-actin (red, phalloidin), G-actin (green, DBP), and nuclei (blue, DAPI). Nuclei are outlined in the white line, based on DAPI staining. Scale bar: 25 μm. **d** Quantification of the average intensity of DBP immunofluorescence in whole osteoclasts. **e** Quantification of the average intensity of DBP immunofluorescence in the nucleus of osteoclasts. **f** Cytochalasin B (10 μM, 10 min)-treated osteoclasts were stained for F-actin using phalloidin (red) and nuclei using DAPI (blue). Scale bar: 25 μm. **g** The size of the SZ in (**f**) per area was scored and statistically compared. Each point represents one cell. *n* = 17–81. *p < 0.05, **p < 0.01, ***p < 0.0001 and ****p < 0.00001 (Student’s *t* test).

Plasticity and dynamics are vital for the function of the SZ, and actin flow is involved in the formation and dynamics of the SZ^32^. The actin-related protein 2/3 (Arp2/3) complex is a pivotal regulator of actin polymerization^14^. GMFB has been identified as a debranching factor that modulates actin dynamics by binding the Arp2/3 complex, inhibiting its function^33^. To further investigate the mechanisms underlying the role of GMFB in SZ formation, we performed double immunofluorescence staining of GMFB and Arp2 on mature osteoclasts. We found that GMFB colocalized with Arp2 in dot-like structures (which represent branch points) in the WT osteoclasts, whereas less dot-like immunostaining for Arp2 was observed in the Gmfb KO osteoclasts (Supplementary Fig. 3a). To determine whether GMFB-mediated actin depolymerization occurred in osteoclasts, we determined the change in actin polymerization dynamics by measuring the G-actin/F-actin ratio at the protein level (Fig. 4a, b). A decreased G-actin/F-actin ratio was obtained regardless of normal conditions or HG treatment, which suggested stronger actin polymerization in the Gmfb KO osteoclasts. The G-actin/F-actin ratio in the Gmfb KO osteoclasts was reversed after overexpression of GMFB in the Gmfb KO osteoclasts, as expected (Supplementary Fig. 3b, c). DBP (vitamin D binding protein) is a marker of G-actin^34^. F-actin and G-actin were assessed using phalloidin (red) and DBP (green) staining in mature osteoclasts to show the distribution and abundance of actin. The results revealed that Gmfb deficiency caused a simultaneous decrease in G-actin content and depletion of nuclear G-actin (Fig. 4c–e). We used an additional antibody, anti-DNase I, which formed a high affinity, stoichiometric 1:1 complex with G-actin^35^, to label G-actin in cells (Supplementary Fig. 3d–f). These results were consistent with DBP staining.

In addition, we used cytochalasin B, a disrupter of actin assembly, to mimic the actin depolymerization function of GMFB in the Gmfb KO osteoclasts. Unexpectedly, 10 μM cytochalasin B treatment for 10 min restricted the SZ size in the Gmfb KO osteoclasts, but the change was not significant (Fig. 4f, g). Additionally, 24 h of 1 μM cytochalasin B treatment reduced the size of the SZ in the WT osteoclasts (Supplementary Fig. 3g, h) and did not expand its size in the Gmfb KO osteoclasts, indicating that long-term, low-dose cytochalasin B treatment had no effect on Gmfb KO osteoclasts. The distinct phenotype with cytochalasin B treatment in the WT and Gmfb KO osteoclasts may be due to the distinct mechanism of cytochalasin B and GMFB on actin polymerization^33,36^. Taken together, these results suggest that Gmfb deficiency negatively regulates SZ formation by decreasing the G/F-actin ratio in actin dynamics.

### Gmfb deficiency increased the nuclear localization of MKL1 and induced MKL1/SRF target gene expression

**Fig. 5:**
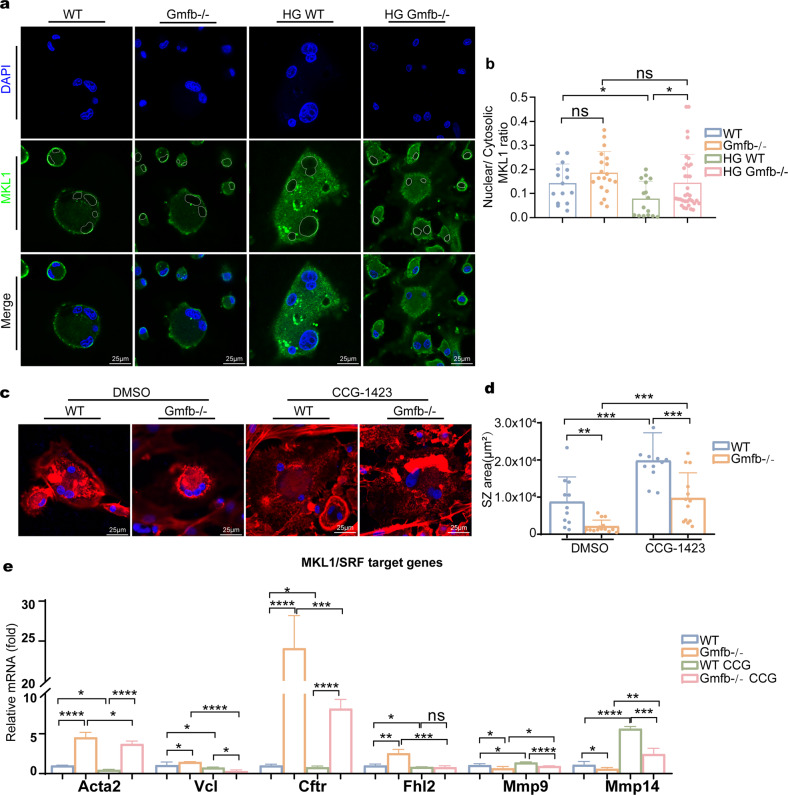
Gmfb deficiency increased the nuclear localization of MKL1 and induced MKL1/SRF target gene expression. **a** Osteoclasts with or without HG were immunostained for MKL1 (green) and nuclei (blue, DAPI). Nuclei are outlined in the white line, based on DAPI staining. Scale bar: 25 μm. **b** Quantification of the nuclear:cytosolic ratio of MKL1 by measuring the average intensity of MKL1 in the nucleus and cytoplasm. **c** Effect of CCG-1423 (1 μM, 24 h) on SZ formation in osteoclasts. Cells were treated with CCG-1423 on day 6 and immunostained for F-actin (red, phalloidin) and nuclei (blue, DAPI). Scale bar: 25 μm. **d** Quantitative analysis of the SZ area in (**c**). Each point represents one cell. **e** Detection of mRNA expression of the MKL1/SRF target genes Acta2, Vcl, Cftr, Fhl2, Mmp14, and Mmp9 by RT‒qPCR. mRNA levels were normalized to Gadph mRNA levels. The data are shown as the mean ± SD (*N* = 2). *p < 0.05, **p < 0.01, ***p < 0.0001 and ****p < 0.00001 (Student’s *t* test).

G-actin accumulates in the nucleus and is involved in transcription^37^. We investigated whether decreased nuclear G-actin in Gmfb deficiency might regulate osteoclast activation-related gene transcription. MKL1 binds to G-actin to mediate its shuttling between the cytoskeleton and nucleus in 3T3 cells^37,38^. Next, we examined MKL1 localization in the osteoclasts treated with HG. MKL1 was detectable in the cytosol and exhibited increased nuclear localization in the Gmfb KO osteoclasts compared to the WT osteoclasts, suggesting that GMFB KO may activate the MKL1/SRF pathway (Fig. 5a, b). Treatment with CCG-1423, a well-established MKL1 inhibitor^39^, resulted in more MKL1-positive puncta in the cytosol (Supplementary Fig. 4) and a larger SZ in both the WT and Gmfb KO rats (Fig. 5c, d), indicating that the MKL1/SRF axis is involved in the regulation of Gmfb-regulated osteoclast activation. We next examined whether Gmfb mediates the osteoclast-related gene expression induced by MKL1/SRF in osteoclasts. Gmfb deficiency upregulated the expression of the MKL1/SRF-dependent target genes Acta2 and Vcl, indicating SRF activation. This effect was blocked by CCG-1423. Moreover, we observed upregulation of Cftr and Fhl2 (anti-osteoclastogenic genes) and downregulation of Mmp9 and Mmp14 (pro-osteoclastogenic genes) in an MKL1/SRF-dependent manner^40–43^ in the Gmfb KO osteoclasts (Fig. 5e). Taken together, our results indicated that Gmfb deficiency inhibited osteoclast hyperactivation by regulating MKL1/SRF-dependent osteoclast activation-related gene expression.

### The GMFB inhibitor DS-30 rescued STZ-induced diabetic osteoporosis

**Fig. 6:**
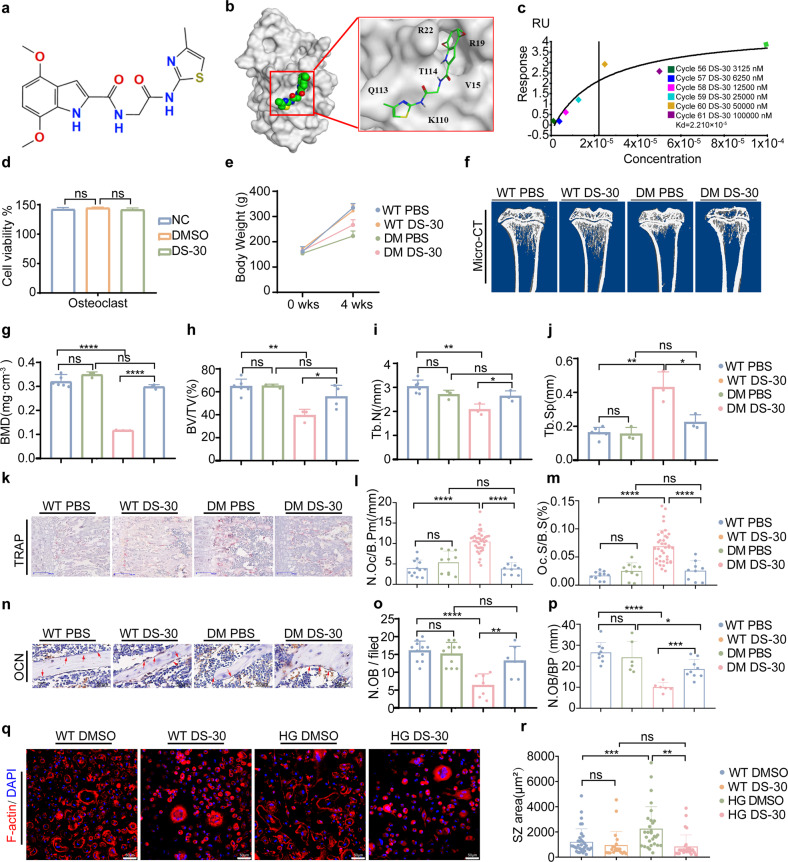
The GMFB inhibitor DS-30 attenuated diabetes-induced osteoporosis in vivo. **a** The chemical structure of DS-30. **b** Molecular docking of DS-30 to GMFB was analyzed in silico. The superimposed structure of DS-30 on the surface of GMFB (gray) was visualized. Different colors in DS-30 correspond to different kinds of chemical bonds with the same color in (**a**). **c** DS-30 binds with GMFB with 2.210 × 10^−5^ binding affinity measured by biocore. **d** Cell viability of osteoclasts treated with DS-30 at a concentration of 50 μM for 24 h was measured by CCK8 assays. **e** Body weight gain in the WT, DS-30-treated WT, diabetic, and DS-30-treated diabetic rats for 4 weeks after STZ induction. **f** Representative micro-CT 3D reconstruction images of proximal tibias. Quantitative analysis of bone morphology, including BMD (**g**), BV/TV (**h**), Tb. N (**i**), and Tb. Sp (**j**). Each point represents one rat. *n* = 4. Data are expressed as the mean ± SD. **k**–**m** TRAP staining of proximal tibial sections was performed to visualize the activated osteoclasts (**k**). Bone histomorphometric analysis for N.Oc/B. Pm (**l**) and OC.S/BS (**m**) in the proximal tibia. Scale bar: 100 μm. Each point represents one cell. *n* = 9–38. Data are expressed as the mean ± SD. **n**–**p** Changes in osteoblasts were observed by immunohistochemistry staining against OCN. Bone histomorphometric analysis of trabecular bone among each group, including osteoblast number per field (N.Ob/per field; **o**) and the number of osteoblasts per bone surface (N.Ob/B. Pm; **p**). Scale bars, 50 μm. Red arrows indicate osteoblasts. *n* = 10–15. **q** Osteoclasts were stained for F-actin using phalloidin (red) and nuclei using DAPI (blue) with or without DS-30 (50 μM) on differentiation day 3. Scale bar: 50 μm. **r** The size of the SZ in (**q**) per area was scored and statistically compared. Each point represents one cell. *n* = 25–35.

Because the Arp2/3 complex plays a key role in nucleating actin filament branching, specific amino acid residues (ARG19, ARG22, and GLN113) in GMFB and the Arp2/3 complex that stabilize the interactions have been identified^28^ and are located mainly in pocket 2 of the 3D structure of GMFB. Thus, we searched for small-molecule drug candidates targeting the binding site of GMFB with Arp2/3 via the protein‒ligand interface fingerprint (PLIF) and ChemDiv using computer virtual screening. Based on the drug accessibility and comprehensive score, DS-30 (4 7-dimethoxy-N~2~-{2-[(4-methyl-1,3-thiazol-2-yl) amino]-2-oxoethyl}-1H-indole-2-carboxamide) was of interest for subsequent intervention experiments (Fig. 6a, b). Biocore analysis revealed the high binding affinity of DS-30 to GMFB in a dose-dependent manner (Fig. 6c). Moreover, 50 μM DS-30 in osteoclasts (Fig. 6d) and two other cell lines (HEK293 and 3T3-L1) (Supplementary Fig. 5) showed no significant cytotoxicity by CCK-8 assays.

To evaluate the effect of DS-30 on T1D-OP, we intraperitoneally injected DS-30 into rats with T1D every day with a maximum concentration in blood of 50 μM. After 4 weeks of administration, the DS-30-treated diabetic rats were heavier than the diabetic rats (Fig. 6e). Importantly, micro-CT analyses showed a recovery of BV/TV, Tb. N, Tb. Th and Tb. Sp in the DS-30-treated diabetic rats, which indicated that DS-30, a small-molecule inhibitor of GMFB, had a therapeutic effect on osteoporosis (Fig. 6f–j).

We next confirmed the effects of DS-30 on osteoclasts. As expected, TRAP staining revealed a decreased number of osteoclasts (Fig. 6k–m), while OCN staining indicated an increased number of osteoblasts (Fig. 6n–p) in the DS-30-treated diabetic histological sections compared to the diabetic histological sections. Furthermore, DS-30 significantly decreased the SZ size under hyperglycemic conditions (Fig. 6q, r). These observations suggest that DS-30 protects against T1D-OP osteoporosis by inhibiting osteoclast hyperactivation with mild cytotoxic effects.

## Discussion

**Fig. 7:**
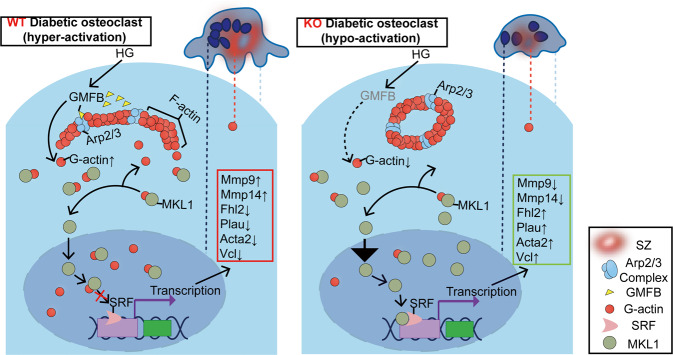
Schematic depicting the function of GMFB in osteoclasts of T1D. In hyperactivated osteoclasts (in a diabetic context), GMFB binds Arp2/3 complex to stimulate filament debranching and inhibit actin nucleation, which are vital for the formation and dynamics of the SZ. Increased G-actin binds to MKL1, blocks MKL1 nucleo-cytoplasmic shuttling thus restrain MKL1/SRF-dependent osteoclast activation-related gene transcription. Gmfb deficiency inhibited osteoclast hyperactivation by limiting SZ enlargement and transcriptionally regulating the expression of anti-osteoclastogenic genes through the G-actin/MKL1/SRF axis.

Clinically, the current treatment of T1D-OP is not evidence-based but is proposed to be similar to that of OP associated with other conditions. Of potentially greater concern, however, is the severe side effects of antiresorbing drugs, including cancer risk, brittle bones and cardiocerebrovascular events^44^. In the present study, we identified GMFB as a novel therapeutic target for T1D-OP. Targeting GMFB substantially ameliorated the phenotype of T1D-OP by restricting SZ enlargement, which is a characteristic of osteoclast hyperactivity. At the molecular level, GMFB, a debranching factor for the Arp2/3 complex, upregulated pro-bone-resorbing gene expression through the G-F actin/MKL1 axis. Finally, the small-molecule inhibitor of GMFB, DS-30, targeting the binding site of GMFB to Arp2/3, was identified and showed dramatic antiosteoporotic effects in rats with T1D. Our work will provide a novel therapeutic strategy for T1D-OP patients, and DS-30 merits further clinical investigation. A working model is proposed in Fig. 7.

Osteoclasts play an important role in bone turnover. Although the function of osteoclasts in T1D is controversial, the present study provided evidence to support the hyperactivity of osteoclasts in T1D, as shown by increased TRAP activity (Fig. 1), larger SZ formation (Fig. 3h, i), upregulated expression of CTSK and RANKL, and downregulated expression of OPG after HG treatment (Fig. 3e). The regulation of SZ formation via podosome organization has been suggested to be imperative for excessive osteoclastic bone resorption and detrimental bone loss^45^. Genetic depletion of SZ components or regulatory proteins, including integrin β3^46^, WASP-associated protein^47^, and Cd44^48^, in mouse models has been shown to be involved in defects in osteoclast function. The Arp2/3 complex was shown to be a major component of the high F-actin density in the podosome core^49^. GMFB binds the Arp2/3 complex to stimulate filament debranching and inhibit actin nucleation^18^. In the present study, we found a decreased level of G-actin (Fig. 4c), a small but dense actin ring structure, in Gmfb KO osteoclasts (Fig. 3h).

The amount of G-actin in cells is limited, and G-actin is the normal substrate for filament formation^50^. When the available G-actin pool is insufficient, it will compromise actin dynamics^51^, which is essential for SZ formation and osteoclast activation. As expected, an enlarged SZ failed to appear in the Gmfb KO osteoclasts in response to HG treatment (Fig. 3h, i). However, we found no significant difference in bone parameters between the WT and KO rats by micro-CT imaging analysis (Fig. 1). We speculated that small but dense actin ring structures in osteoclasts might still support bone resorption to some degree to maintain normal bone remodeling under physiological conditions.

Cytochalasin B is a well-studied inhibitor of actin polymerization. This molecule directly binds F-actin, blocks the addition of G-actin to the growing end of the polymer chain, and then shortens actin filaments^36^. GMFB directly binds the Arp2/3 complex, but not actin, to promote actin depolymerization^18^. In our study, the F-actin staining pattern in the osteoclasts treated with cytochalasin B appeared as a small punctate or diffuse pattern throughout the cytoplasm (Fig. 4f). Such a punctate pattern of F-actin staining is considered to almost correspond to that of G-actin^34^. However, a substantially less punctate pattern of F-actin was observed in the Gmfb KO osteoclasts after cytochalasin B treatment, indicating that Gmfb KO-dependent actin nucleation was stronger than actin depolymerization of cytochalasin B due to their distinct mechanism in actin depolymerization.

Emerging evidence has shown that a large portion of G-actin accumulates in the nucleus and is involved in transcription, chromatin remodeling, and RNA polymerase functions^52^. Our immunofluorescence results clearly showed that GMFB deficiency prevented the nuclear localization of G-actin. G-actin mediates MKL1 shuttling between the nucleus and cytoplasm. MKL1 was segregated in the cytoplasm by binding of G-actin to the N-terminal RPEL motifs of MKL1, which occludes the bipartite nuclear localization signal (NLS). This dimer form also facilitates CRM1-mediated MKL1 nuclear export^47^. When Rho activation or other signals decrease the amount of G-actin, MKL1 is liberated to the nucleus, where it interacts with and activates SRF, thus initiating an MKL1/SRF-dependent gene expression program^53^. Acta2 and Vcl are direct MKL1/SRF-dependent targets in different cells^37,54^. The upregulation of Acta2 and Vcl in the Gmfb KO osteoclasts indicated that GMFB negatively regulated MKL1/SRF activity. In addition, other MKL1/SRF-regulated genes that promote bone resorption, including Cftr and Fhl2, showed decreased levels in the Gmfb KO osteoclasts (Fig. 5e). Thus, GMFB may transcriptionally regulate pro-bone resorption gene expression in osteoclasts via the G-actin/MKL1/SRF pathway.

Osteomorphs were first reported in 2021, and fission products were shown to refuse with other osteoclasts in osteoclast recycling^31^. Compared with that of the WT group, more osteomorph formation in the Gmfb KO osteoclasts was observed. Because the osteomorphs showed shallower pit resorption^31^, we speculated that under normal conditions, osteomorphs, as well as SZ-bearing osteoclasts, collaboratively function in bone remodeling to maintain bone homeostasis in Gmfb KO rats, partially indicating that there was no significant bone remodeling phenotype change between the WT and KO rats.

Based on our findings above that the interaction between GMFB and Arp2/3 plays a crucial role in regulating osteoclast activity and that three binding sites (ARG19, ARG22, and GLN113) of GMFB to Arp2/3 have been identified^28^, computer virtual screening with the ChemDiv library was performed by targeting the three binding sites. DS-30 was identified with a high score by comprehensive evaluation. As expected, administration of DS-30 effectively inhibited SZ enlargement and alleviated osteoporosis in rats with T1D.

In conclusion, using a systemic KO approach, we demonstrated that Gmfb deficiency significantly improved the phenotype of T1D-OP by suppressing osteoclast hyperactivation. The protective effect of Gmfb deficiency was mediated by limiting SZ enlargement and transcriptionally regulating the expression of osteoclast activation-related genes through the G-actin/MKL1/SRF axis. GMFB intervention may represent a novel therapeutic strategy for T1D-OP. Small molecule inhibitors of GMFB will shed light on translational research in T1D-OP.

### Limitations of study

First, we used a systemic KO rat model rather than osteoclast-specific KO in this study. We cannot exclude the effect of Gmfb KO in other cell types in bone remodeling, including bone marrow mesenchymal stem cells, osteoblasts, osteocytes, and even adipocytes in a diabetic context. Likewise, in the intraperitoneal administration of DS-30 in rats with T1D, we failed to exclude the effect of DS-30 on other cells. Cell‒cell communication in bone remodeling deserves further exploration. A cell type-specific KO strategy might help us reveal the accurate mechanism of GMFB in osteoclasts of T1D-OP. Second, although our data in Gmfb KO osteoclasts support the activation of the MKL1/SRF pathway, which is well established in 3T3 cells^53^, the intervention of MKL1 or SRF using the knockdown/knockout technique is required in osteoclasts. These issues merit further investigation.

## Supplementary information

Supplemental material
