# Supplementary material for: Glia maturation factor beta deficiency protects against diabetic osteoporosis by suppressing osteoclast hyperactivity
Source: Exp Mol Med. 2023 May 1;55(5):898–909. doi: 10.1038/s12276-023-00980-8 (PMC10238439; doi:10.1038/s12276-023-00980-8)
Supplement: Supplementary file 1 — Supplemental material [file 12276_2023_980_MOESM1_ESM.docx]

| arget gene | Sequences (5’→3’) |
| --- | --- |
| Rat Gmfb-Forward | CTAAGCTCTGCGGAGCCAAGG |
| Rat Gmfb-Reverse | GGCAAATGCAGCAGGTGGAG |
| Rat Nfatc1-Forward | CAGGCTACAGCCGCAGTAAA |
| Rat Nfatc1-Reverse | TTGCTGAAGCGTGGTTGTC |
| Rat Ctsk-Forward | TCCTCAACAGTGCAAGCGAA |
| Rat Ctsk-Reverse | CCAGCGTCTATCAGCACAGA |
| Rat OPG-Forward | TGGCACACGAGTGATGAATGCG |
| Rat OPG-Reverse | GCTGGAAAGTTTGCTCTTGCG |
| Rat Rankl-Forward | CATCGGGTTCCCATAAAG |
| Rat Rankl-Reverse | GAAGCAAATGTTGGCGTA |
| Rat Fbxo7-Forward | CTTCAAAAGCGGACCCAGCC |
| Rat Fbxo7-Reverse | CCAGTGAGGGCATCCTTGTT |
| Rat Vcam1-Forward | ACTGTGACCTGTCAGCGAAG |
| Rat Vcam1-Reverse | TTAGGGACCGTGCAGTTGAC |
| Rat CD11B-Forward | ATGACCACCTCCTGCTTGTG |
| Rat CD11B-Reverse | CTGCCCACAATGAGTGGTACA |
| Rat CCR2-Forward | GGGCTGTGAGGCTCATCTTT |
| Rat CCR2-Reverse | GGCCTGGTCTAAGTGCATGT |
| Rat CD47-Forward | AGAGAATCATTCTGCTGCTGGTTGC |
| Rat CD47-Reverse | TGGTGAAAGAGGTCATTCCAAAAGC |
| Rat OC-Stamp-Forward | GAGTGCTGGGCTGTGTTACT |
| Rat OC-Stamp-Reverse | ACCTCAAATGTCAGGCTCCG |
| Rat DC-Stamp-Forward | ATCGGCTCATTTCCTCCGTG |
| Rat DC-Stamp-Reverse | ACACTGAGCCGTGGTTTAAGA |
| Rat Acta2-Forward | GGATCAGCGCCTTCAGTTCT |
| Rat Acta2-Reverse | CAGGGCTAGAAGGGTAGCAC |
| Rat Vcl-Forward | AAGGAGGCAAAAGGAAAC |
| Rat Vcl-Reverse | GAAAGAAGAGGCAGAAAACA |
| Rat Cftr-Forward | AGCAAATGACATCACCTCAGGA |
| Rat Cftr-Reverse | ACAGGTTCAGGTTGGACTCG |
| Rat Fhl2-Forward | CTGAACGCTTTGACTGCCAC |
| Rat Fhl2-Reverse | CTCCTCACAGGTGTTGGCAT |
| Rat Mmp14-Forward | TCGGAGGGGATACCCACTTT |
| Rat Mmp14-Reverse | GGAGGGGTCGTTGGAATGTT |
| Rat Mmp9-Forward | GATCCCCAGAGCGTTACTCG |
| Rat Mmp9-Reverse | GTTGTGGAAACTCACACGCC |
| Rat GADPH-Forward | TGGCCTCCAAGGAGTAAGAAAC |
| Rat GADPH-Reverse | GGCCTCTCTCTTGCTCTCAGTATC |

**Table. S1 Primer sequences for RT-qPCR**


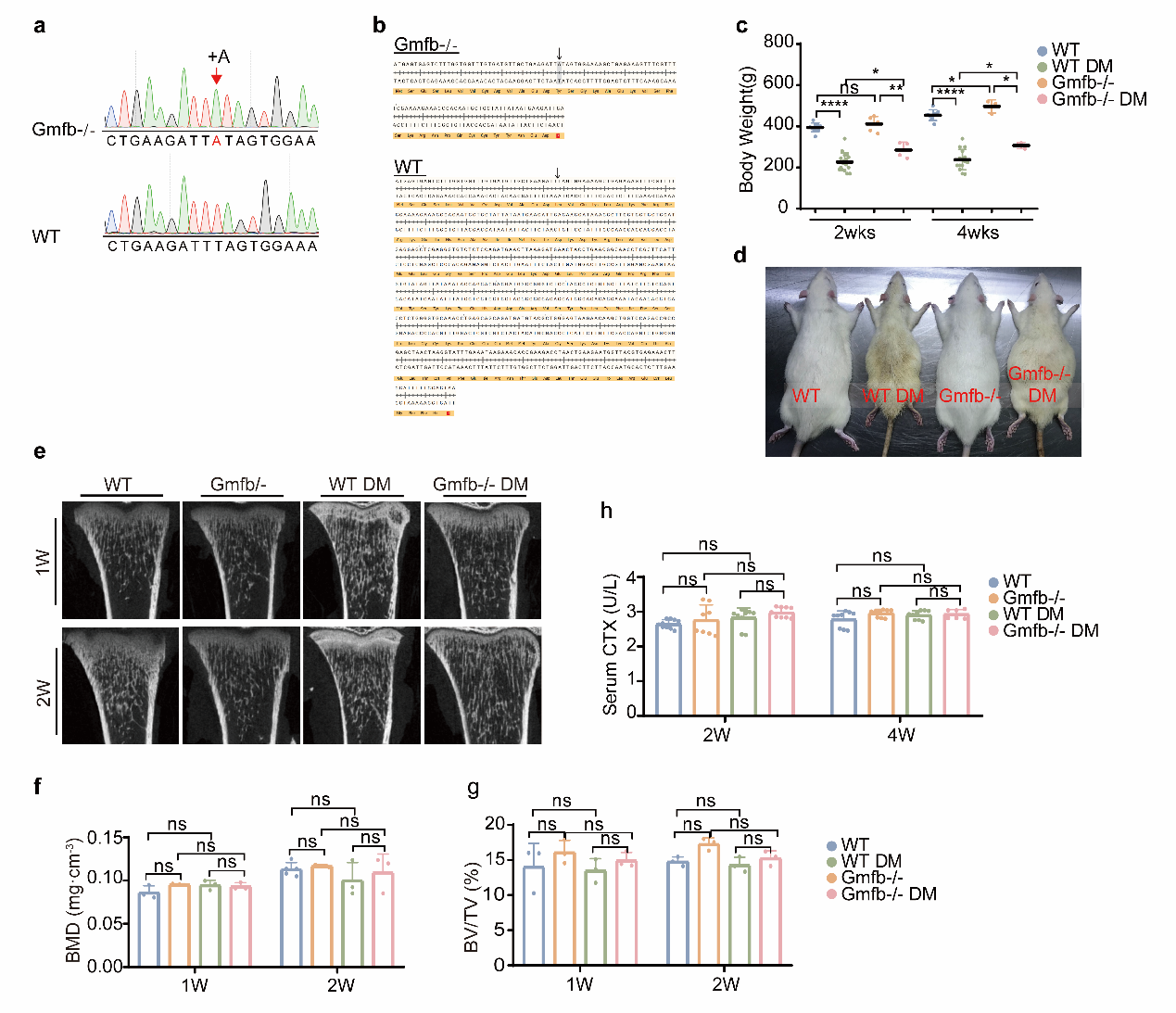


**Fig. S1:** Gmfb deficiency regulated **overt phenotypic features**in T1DM rats***.* a One base (a) was inserted into the sequence of g*mfb* gene on exon 2, resulting in the early termination of GMFB protein translation. b The amino acids corresponding to the full-length(below) and truncated(upper) GMFB protein sequences. c Body weight gain in WT and Gmfb KO rats induced or not induced by STZ. d Body size and hair color of WT and Gmfb KO rats induced or not induced by STZ.** Each point represents one rat. **n=7-10. e The representative micro-CT 2D reconstruction images of proximal tibias. f-g Quantitative analysis of bone morphology including bone mineral density (BMD) (f), bone volume per total volume (BV/TV) (g).** Each point represents one rat. n=3. **h** Serum concentrations of CTX-1 detected in WT and Gmfb KO rats induced or not induced by STZ by ELISA. Data are presented as the mean ± SD of three independent experiments. * p < 0.05, ** p < 0.01, *** p < 0.0001 and **** p < 0.00001 (Student’s t test).


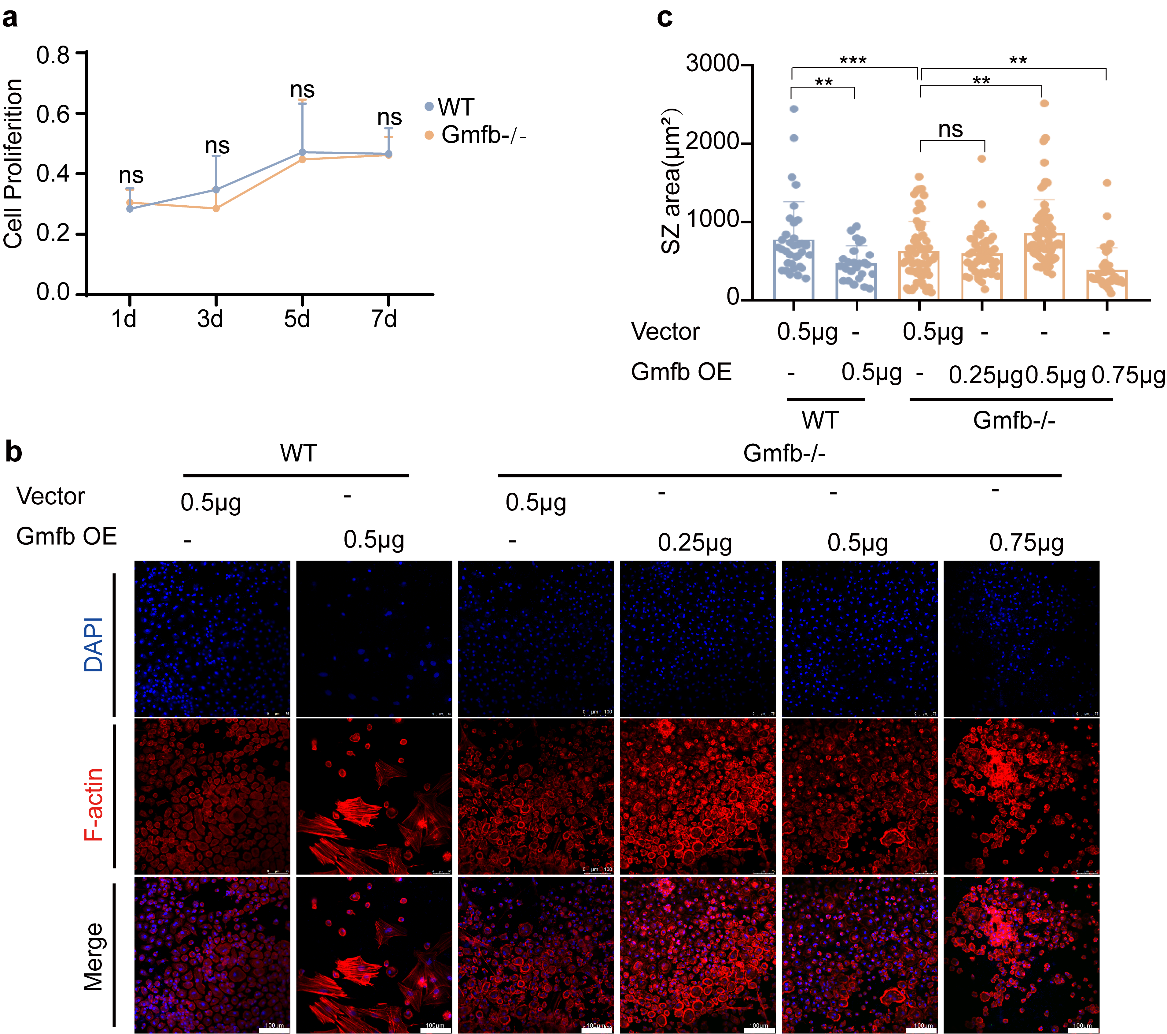


**Fig. S2: Overexpression of Gmfb rescue SZ formation in Gmfb KO osteoclasts. a Cell proliferation was measured by EdU Cell Proliferation Kit with TMB. n=4. b Osteoclasts were transient transfected pcDNA3.1(+) or Gmfb-pcDNA3.1(+) on the fourth day of differentiation were immunostained for F-actin (red, phalloidine), and nuclei (blue, DAPI). Scale bar: 75μm. c Quantitative analysis of SZ area during osteoclastogenesis in (b).** Each point represents one cell. **n=25-67.** * p < 0.05, ** p < 0.01, *** p < 0.0001 and **** p < 0.00001 (Student’s t test).


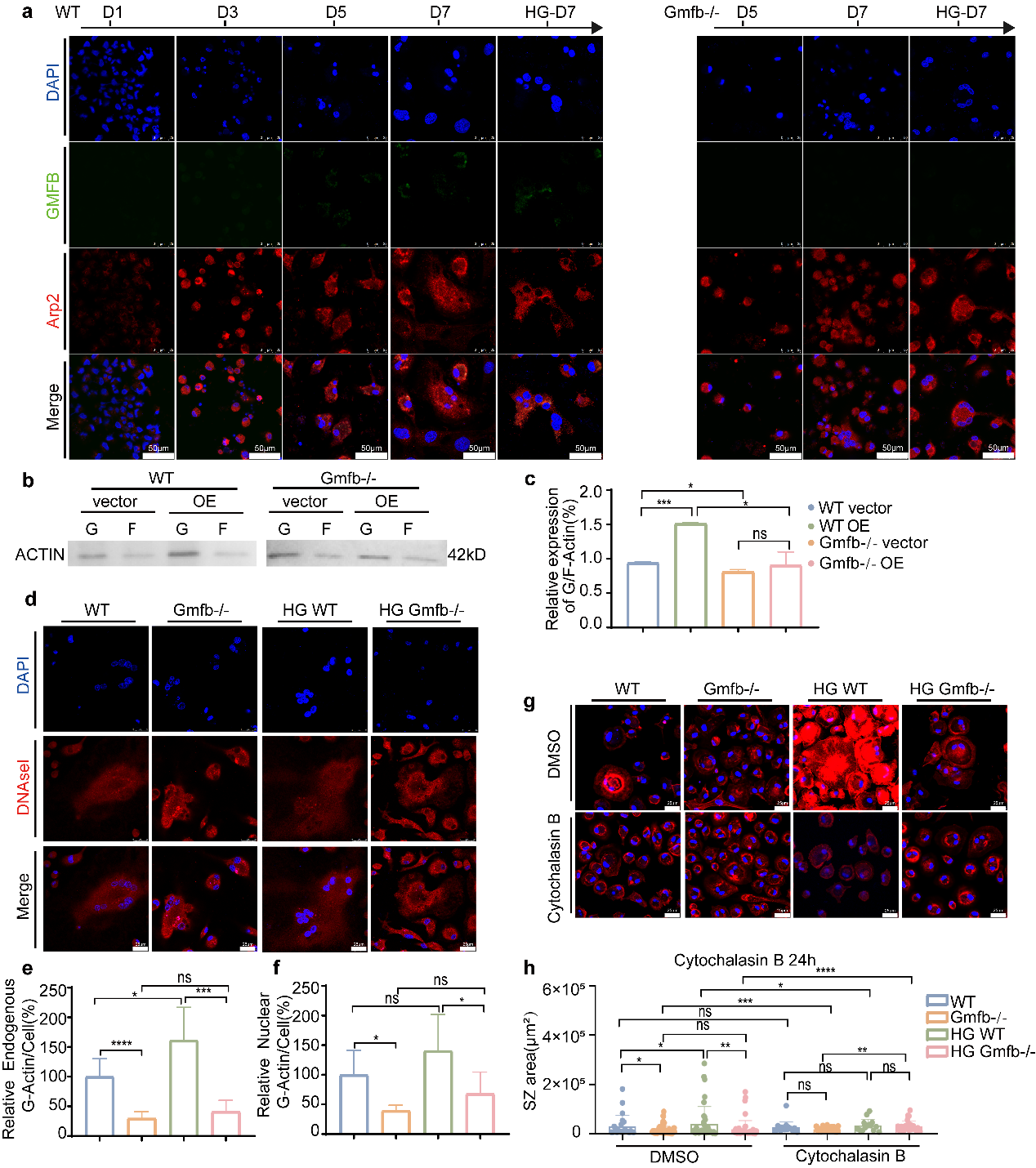


**Fig. S3: GMFB was involved in actin depolymerization through Arp2/Arp3 complex. a Osteoclasts from WT rats treated with differentiation medium for 1d, 3d, 5d, and 7d and osteoclasts from Gmfb-/- rats treated with differentiation medium for 5d and 7d were immunostained for Arp2 (red), GMFB (green) and nuclei (blue, DAPI). Scale bar: 25μm. b-c Western blot quantification of the G/F actin ratio within cells which transient transfected pcDNA3.1(+) or Gmfb-pcDNA3.1(+) on the fourth day of differentiation. The data are shown as the mean ± SD (N = 3)**. **d WT and Gmfb KO osteoclasts treated with HG were immunostained for DNaseI to detect G-actin (green), and nuclei (blue, DAPI). Scale bar: 25μm. e Quantification of the average intensity of DNaseI immunofluorescence in whole osteoclasts. f Quantification of the average intensity of DNaseI immunofluorescence in the nucleus of osteoclasts.** Each point represents one cell. **n=15-21. g Cytochalasin B (1μM, 24h) treated osteoclasts were stained for F-actin using phalloidin(red) and nuclei using DAPI (blue). Scale bar: 25μm. h The size of SZ in (g) per area were scored and statistically compared. Scale bar: 25μm.** Each point represents one cell. n=8-20. Data are expressed as mean±SD. * p < 0.05, ** p < 0.01, *** p < 0.0001 and **** p < 0.00001 (Student’s t test).


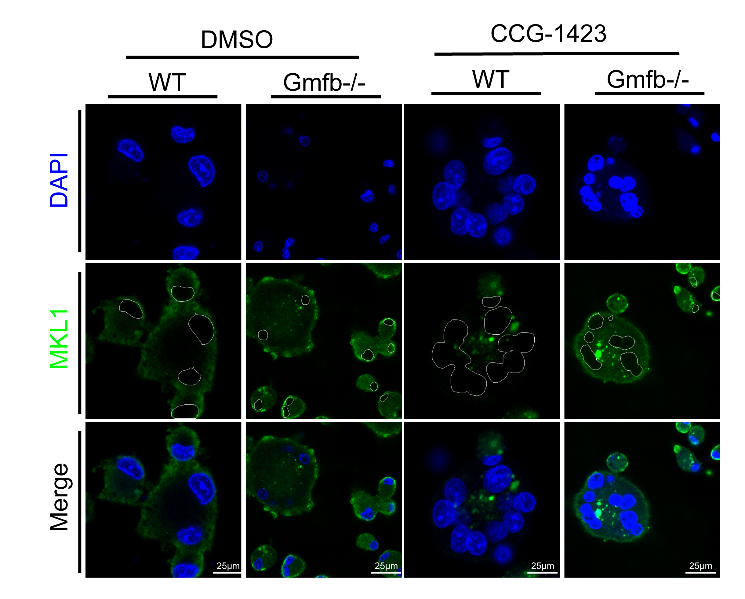


**Fig. S4: Osteoclasts treated or not treated with CCG-1423 were immunostained for MKL1 (green) and nuclei (blue, DAPI).**


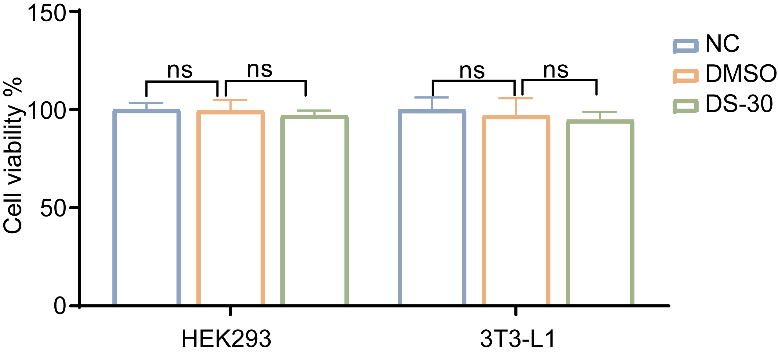


**Fig. S5:** Cell viability of HEK293 and 3T3-L1 which treated with DS-30 at a concentration of 50μM for 24h was measured by CCK8 assay.
